# Mendelian randomization and genetic colocalization infer the effects of the multi-tissue proteome on 211 complex disease-related phenotypes

**DOI:** 10.1186/s13073-022-01140-9

**Published:** 2022-12-12

**Authors:** Chengran Yang, Anne M. Fagan, Richard J. Perrin, Herve Rhinn, Oscar Harari, Carlos Cruchaga

**Affiliations:** 1 Department of Psychiatry, Washington University School of Medicine, 4444 Forest Park Ave., Box 8134, St. Louis, MO 63108 USA; 2 NeuroGenomics and Informatics Center, Washington University School of Medicine, St Louis, MO USA; 3 Hope Center for Neurological Disorders, Washington University School of Medicine, St. Louis, MO USA; 4 Department of Neurology, Washington University School of Medicine, St. Louis, MO USA; 5 The Charles F. and Joanne Knight Alzheimer’s Disease Research Center, Washington University School of Medicine, St. Louis, MO USA; 6 Department of Pathology and Immunology, Washington University School of Medicine, St. Louis, MO USA; 7 Department of Bioinformatics, Alector, Inc., 151 Oyster Point Blvd. #300, South San Francisco, CA USA

**Keywords:** Protein quantitative trait loci, Mendelian randomization, Genetic colocalization, Multi-tissue proteomics, Complex human phenotypes

## Abstract

**Background:**

Human proteins are widely used as drug targets. Integration of large-scale protein-level genome-wide association studies (GWAS) and disease-related GWAS has thus connected genetic variation to disease mechanisms via protein. Previous proteome-by-phenome-wide Mendelian randomization (MR) studies have been mainly focused on plasma proteomes. Previous MR studies using the brain proteome only reported protein effects on a set of pre-selected tissue-specific diseases. No studies, however, have used high-throughput proteomics from multiple tissues to perform MR on hundreds of phenotypes.

**Methods:**

Here, we performed MR and colocalization analysis using multi-tissue (cerebrospinal fluid (CSF), plasma, and brain from pre- and post-meta-analysis of several disease-focus cohorts including Alzheimer disease (AD)) protein quantitative trait loci (pQTLs) as instrumental variables to infer protein effects on 211 phenotypes, covering seven broad categories: biological traits, blood traits, cancer types, neurological diseases, other diseases, personality traits, and other risk factors. We first implemented these analyses with cis pQTLs, as cis pQTLs are known for being less prone to horizontal pleiotropy. Next, we included both cis and trans conditionally independent pQTLs that passed the genome-wide significance threshold keeping only variants associated with fewer than five proteins to minimize pleiotropic effects. We compared the tissue-specific protein effects on phenotypes across different categories. Finally, we integrated the MR-prioritized proteins with the druggable genome to identify new potential targets.

**Results:**

In the MR and colocalization analysis including study-wide significant cis pQTLs as instrumental variables, we identified 33 CSF, 13 plasma, and five brain proteins to be putative causal for 37, 18, and eight phenotypes, respectively. After expanding the instrumental variables by including genome-wide significant cis and trans pQTLs, we identified a total of 58 CSF, 32 plasma, and nine brain proteins associated with 58, 44, and 16 phenotypes, respectively. For those protein-phenotype associations that were found in more than one tissue, the directions of the associations for 13 (87%) pairs were consistent across tissues. As we were unable to use methods correcting for horizontal pleiotropy given most of the proteins were only associated with one valid instrumental variable after clumping, we found that the observations of protein-phenotype associations were consistent with a causal role or horizontal pleiotropy. Between 66.7 and 86.3% of the disease-causing proteins overlapped with the druggable genome. Finally, between one and three proteins, depending on the tissue, were connected with at least one drug compound for one phenotype from both DrugBank and ChEMBL databases.

**Conclusions:**

Integrating multi-tissue pQTLs with MR and the druggable genome may open doors to pinpoint novel interventions for complex traits with no effective treatments, such as ovarian and lung cancers.

**Supplementary Information:**

The online version contains supplementary material available at 10.1186/s13073-022-01140-9.

## Background

Two-sample Mendelian randomization (MR) [1, 2], a genetic epidemiological method, has been increasingly used to infer the causal effect of an exposure on an outcome using genetic variants as instrumental variables (IVs) from the summary statistics of the human disease-related phenotypes. Colocalization approaches [3–5] have been used to support inference by reducing the likelihood that linkage disequilibrium (LD) affected the MR findings [6, 7]. As proteins are more likely to be used as drug targets than other molecular traits [8, 9], MR analyses accompanied with colocalization using pQTLs as IVs would be valuable for the broad community of human genetics [10]. There were multiple MR studies inferring the effects of proteins on diseases, but most of them focused on fewer than 10 diseases [6, 11–13].

In 2020, Zheng and colleagues [7] performed a study of phenome-wide two-sample MR and colocalization on over 200 phenotypes (diseases/risk factors) using 1002 proteins from five large-scale plasma pQTL datasets. A similar proteome-by-phenome-wide MR study by [7, 14] and colleagues [14] used 64 plasma pQTLs as IVs. However, these two studies were based exclusively on plasma proteomics, limiting its application to other disease-relevant tissues [15], such as the brain when investigating psychiatric or neurological diseases or CSF when investigating neuroimmune-related disorders. In our previous study [16] using a cohort including Alzheimer disease (AD) cases and cognitively normal individuals, we demonstrated that pQTLs (~ 20% cis pQTLs; ~ 80% trans pQTLs) are tissue-specific. In the same study, we performed sensitivity analyses demonstrating that the pQTLs from all three tissues (CSF, plasma, and brain) were neither disease- nor age-specific, suggesting that these multi-tissue pQTLs could be used as instrumental variables for MR analyses to identify potential causal proteins for multiple disease-related traits, not limited to neurological diseases.

**Fig. 1:**
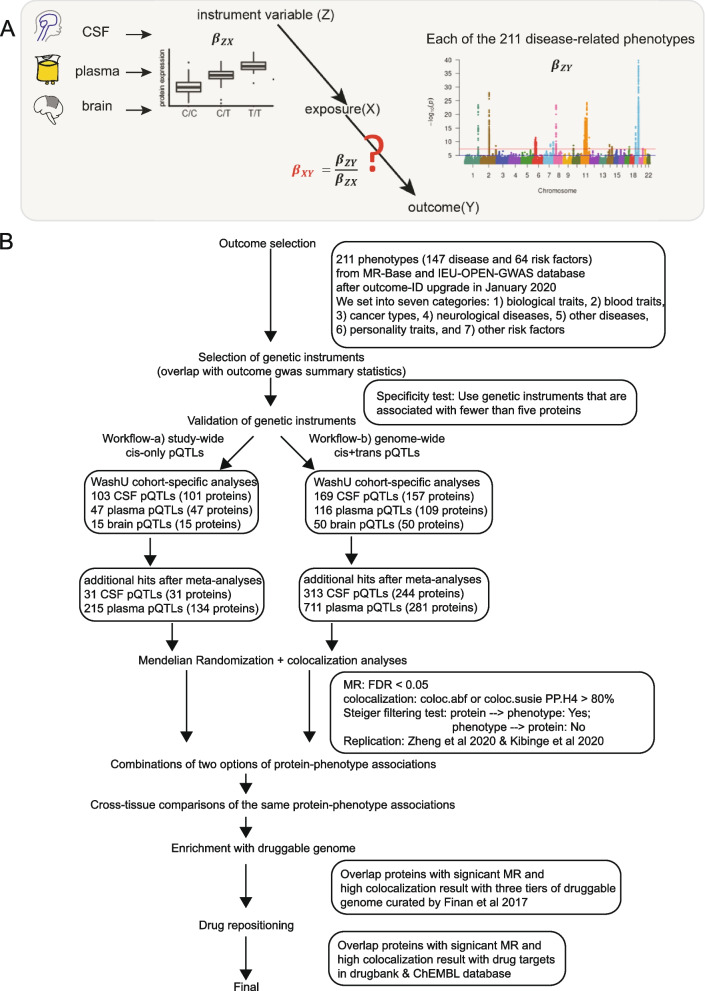
Schematics of the study design and flowchart of analyses performed in this study. **A** Instrumental variables were selected from multi-tissue pQTL datasets and used for performing Mendelian randomization with 211 disease-related phenotypes. **B** In summary, eight steps were implemented in this study: step 1 as outcome selection; step 2 as genetics instrumental variables selection; step 3 as validation of genetic instrumental variables; step 4 as MR and colocalization analyses with two workflows—(4a) *cis*-only instrumental variables passing study-wide significance and (4b) *cis*- and *trans*-instrumental variables passing genome-wide significance; step 5 as combinations of workflows of protein-phenotype associations within each tissue; step 6 as cross-tissue comparisons of the same protein-phenotype associations; step 7 as enrichment with druggable genome; and step 8 as drug repositioning

Here, we leverage this large multi-tissue pQTL atlas, as well as additional CSF and plasma pQTL datasets to identify novel MR-prioritized proteins for 211 complex disease-related phenotypes [7] (Fig. 1, Additional file 2: Table S1), which include 37 biological traits, 21 blood traits, 23 cancer types, 18 neurological diseases, 94 other diseases (defined as any other diseases that are not cancers or neurological diseases), 10 personality traits, and eight other risk factors (such as fractured bone sites: ankle/arm/wrist, flatulence). We expand our prior study from using neurological traits to over 200 complex phenotypes [16]. We complement the previous single-tissue findings: plasma-only [7, 14] or brain-only proteome [6], with a multi-tissue approach. We highlight the findings from MR, druggable genome, and drug repurposing in a tissue-dependent manner.

## Methods

### Washington University proteomic data QC process

A multiplexed, aptamer-based platform [17] was used to measure the relative concentrations (relative fluorescent units) of proteins from CSF, plasma, and brain tissues, using 1305 modified aptamers in total. The assay covers a dynamic range of 10^8^ and measures all three major categories: secreted, membrane, and intracellular proteins.

Aliquots of 150 μl of tissue were sent to the Genome Technology Access Center at Washington University in St. Louis for protein measurement. Assay details have been previously described by Gold and colleagues [17] from SomaLogic Inc. In brief, modified single-stranded DNA aptamers are used to bind the target proteins that are then quantified in the DNA microarray format. Protein abundances are quantified as relative fluorescence units.

Quality control (QC) was performed at both levels of samples and aptamers using the controlling aptamers (positive and negative controls) and calibrator samples. At the sample level, hybridization controls from each plate were used to correct for systematic variability in hybridization. The median signal from all aptamers was used to correct for within-run technical variability. This median signal was further assigned to different dilution sets within each tissue. For CSF and brain samples, a 20% dilution rate was used. For plasma samples, three different dilution rates (40%, 1%, and 0.005%) were used.

To QC the proteomics datasets, the protein outliers were first removed by applying the first four steps: (1) Minimum detection filtering. The limit of detection was defined as the summation of the average expression level of the new NP-buffer (used as dilution buffer of CSF samples from plate-42 to plate-50) and twofold standard deviation. If the protein for a given sample was below the limit of detection, this sample was an outlier. Collectively, if the number of outliers given a protein was greater than 15% of the total sample size, the analyte was removed. (2) Flagging proteins based on the scale-factor difference. The scale factor difference was calculated as the absolute value of the maximum difference between the calibration scale factor per protein and the median for each of the plates run. If the value for this protein was greater than 0.5, the protein failed this criterion. (3) Coefficient of variation of calibrators filtering. The coefficient of variation for each aptamer was calculated by dividing the standard deviation by the average of each calibrator at the raw protein level. If the protein had a coefficient of variation of greater than 0.15, this analyte failed this QC step. (4) Interquartile range (IQR) strategy. Outliers were identified if the sample was located outside of either end of distribution using a 1.5-fold IQR given the log10 transformation of the protein level. Collectively, if the number of outliers given a protein was greater than 15% of the total sample size (or non-outliers given a protein was fewer than 85% of the total sample size), this protein was filtered. Proteins were kept after passing all four criteria above for all the downstream statistical analysis.

An orthogonal approach was used to determine sample outliers based on IQR. Collectively, if the number of outliers given a protein was greater than 15% of the total number of proteins that passed QC (or non-outliers given an analyte was fewer than 85% of the total number of proteins passed QC), this sample was labeled as an outlier. Furthermore, the analytes were removed if shared by most (~ 80%) of the subject outliers. After this second removal of analytes, sample outliers were called again. Sample outliers were again removed.

### WashU multi-tissue pQTL summary statistics

#### Overview

The summary statistics for pQTL for three tissues from the Washington University cohort were processed as described in the publication of Yang et al. [16] and publicly available at the National Institute on Aging Genetics of Alzheimer’s Disease Data Storage Site (NG00102 dataset: https://www.niagads.org/datasets/ng00102). In brief, we performed a linear regression on genotype dosage (additive model) against each protein level measured by an aptamer-based platform [17], including age, sex, first two principal component (PC) factors from population stratification, and genotype platforms as covariates. We generated a dataset in three tissues (835 CSF, 529 plasma, and 380 brain), by profiling thousands of proteins (713 CSF, 931 plasma, and 1079 brain) and 14,059,245 imputed and directly genotyped common variants (variants with minor allele frequency (MAF) ≥ 2% were kept per genotype QC). CSF samples were collected the morning after an overnight fast, processed, and stored at − 80 °C. Plasma samples were collected the morning after an overnight fast, immediately centrifuged, and stored at − 80 °C. Brain tissues were collected from fresh frozen human parietal lobes. Disease status was defined per the Clinical Dementia Rating Scale at the time of lumbar puncture (CSF) or blood draw (plasma). For brain samples, status was defined per the postmortem neuropathological analysis of study participant brains based on the criteria of Consortium to Establish a Registry for AD and/or Khachaturian. Hereafter, we termed the dataset as Washington University (WashU) cohort [16].

#### Disease-specific analyses

To detect if there is a disease-specific effect on pQTLs, we performed linear regression on the same pQTL sets identified from the above default model using three additional models: (i) joint analysis including disease status as another covariate (cognitive unimpaired controls versus non-controls), (ii) AD case only using the same covariates as the default model, and (iii) cognitive unimpaired controls only using the same covariates as the default model (see Extended Data Figure 4 from [16]). Overall, we observed all comparisons were significantly highly correlated, indicating no disease-specific pQTLs.

#### Age-specific analyses

To detect if there is an age-specific effect on pQTLs, we performed separate analyses in participants younger and older than the average age of our cohort and compared the regression coefficient of all the significant pQTLs from the above default model to identify any age-specific effect. Overall, we observed all comparisons were significantly highly correlated, indicating no age-specific pQTLs.

#### Power analysis of pQTLs

To investigate the power-sample size relationship of the pQTL datasets, we used the function powerEQTL.SLR() from the powerEQTL R package [18] to estimate the sample size given a fixed MAF (as 0.2), effect size, and variance at the power values range from 0.2 to 0.8. The median of effect size and variance were learned from the pQTL used in MR analyses within each tissue (Additional file 1: Fig. S1).

### Meta-analyses with other studies

#### CSF pQTLs

We performed fixed-effect meta-analyses on two CSF pQTL studies using METAL [19] based on inverse-variance weighting. The two studies used for meta-analysis on CSF pQTLs were from Parkinson’s Progression Markers Initiative (PPMI) released in 2019 [20] (*N* = 132) and from the WashU cohort [16]. We included 709 CSF proteins shared in both studies. We did not include all proteins in the meta-analyses because of the assay differences (WashU used the 1305 version from the SOMAscan assay; PPMI2019 used the Merck-customized version from the SOMAscan assay).

For the cohort of the PPMI release in 2019, 132 samples with European ancestry passed proteomic QC in this release. The PPMI cohort comprised both PD and healthy participants with their clinical, imaging, and biospecimen biomarker assessment at 21 clinical sites since it launched in the year of 2010 [20]. The pQTL dataset was generated in-house. We performed a linear regression (additive model), including age, sex, and the first two PC factors from population stratification as covariates.

#### Plasma pQTLs

We performed fixed-effect meta-analyses on three plasma pQTL studies using METAL [19] based on inverse-variance weighting. The three studies used for meta-analysis on plasma pQTLs were from the INTERVAL cohort [21] (*N* = 3301), the SCALLOP cohort [22] (*N* = 30,931), and the WashU cohort [16]. For meta-analyses on WashU and INTERVAL, we included 746 plasma proteins shared in both studies. For meta-analyses on WashU, INTERVAL, and SCALLOP, we included 49 plasma proteins shared in three studies. We did not include all proteins in the meta-analyses because of the assay differences (WashU used 1305 version from the SOMAscan platform; INTERVAL used the University of Cambridge-customized version from the SOMAscan platform; SCALLOP used 92 cardiovascular proteins from the OLINK assay. All three studies used different assays/platforms, either the versions of the same technology or the differences in technologies).

For the cohort of INTERVAL, we downloaded the pQTL summary statistics from the publication by Sun and colleagues in 2018 [21]. The sample size used for the pQTL mapping of this cohort was 3301. The participants of the INTERVAL cohort were from 25 sites of England’s National Health Service Blood and Transplant from 2012 to 2014.

For the cohort of SCALLOP, we downloaded the pQTL summary statistics from the publication by Folkerson and colleagues in 2020 [22]. The sample size used for the pQTL mapping of this cohort was 30,931. The SCALLOP cohort consisted of 15 studies of European ancestry, and each study performed its own pQTL mapping. Meta-analysis was performed using all 15 studies.

#### Heterogeneity of pQTLs

We checked the heterogeneity of pQTLs (HetPVal from two schemes of METAL [19]) across studies from METAL and found the majority (92% for cis-only and 100% for cis + trans) of pQTLs used as IVs are not heterogeneous (HetPval ≥ 0.05). We added the flags on the MR results using these heterogeneous meta-analyzed pQTLs with extra columns “flag_heterogeneity” (TRUE if pQTL has a HetPVal < 0.05 under the scheme STDERR), “flag_heterogeneity2” (TRUE if pQTL has a HetPVal < 0.05 under the scheme SAMPLESIZE), and “flag_hetero.all” (TRUE if both heterogeneity flags are TRUE).

### Human phenotype selection

We focused on the human phenotypes from a prior study [7] that aggregated more than 200 phenotypes for MR analyses. The authors selected complex human phenotypes using the MR-Base database [2] and using two criteria: (1) the GWAS with the largest sample size given the same disease with multiple GWAS and (2) GWAS with full summary statistics available as feasible to perform downstream analyses. Diseases and risk factors were chosen as outcomes. Starting with 225, we found, however, only 211 phenotypes with a valid ID (Fig. 1A, Additional file 2: Table S1) due to the upgrade for the database of MR-Base [2] and its accompanied MRC Integrative Epidemiology Unit OpenGWAS project [23] since January 2020. The summary statistics for all phenotypes were downloaded from the Integrative Epidemiology Unit OpenGWAS project website as VCF files [24] and corresponding index files for later colocalization usage. We categorized these phenotypes into seven groups: (1) biological traits, (2) blood traits, (3) cancer, (4) neurological diseases, (5) other diseases and traits, (6) personality traits, and (7) other risk factors. LD score regression [25] v1.0.1 was used to calculate the genetic correlations between each pair of phenotypes.

### Instrumental variable selection for MR

Two workflows (Fig. 1B) were used to select IVs: Workflow-a cis study-wide significant pQTL (termed as “cis-only” analysis) after keeping variants associated with fewer than five proteins (note: variants within the same LD block with the sentinel variants in the pleiotropic regions were also removed). This was performed to avoid pleiotropic effects as in a previous study [7] and also based on the empirical distribution of IVs overlapped with pleiotropic variants identified from all three tissues across different protein thresholds (Additional file 1: Fig. S2). Multiple testing significance thresholds were defined as protein-variant pairs with a *p-*value below the threshold of (5 × 10^−8^/number-principal-components). The number of PCs was derived as the minimum principal component number that cumulatively explains 95% of the total proteomic variance within each tissue after QC. For proteomics of CSF, plasma, and brain tissues, the number of PCs was 169, 230, and 75, respectively. Thus, the *p*-value thresholds were 3 × 10^−10^, 2 × 10^−10^, and 7 × 10^−10^, respectively. Workflow-b both cis and trans genome-wide significant pQTLs (*p*-value < 5 × 10^−8^), termed as “cis + trans” analysis after removing highly pleiotropic variants, the same as workflow-a. We next performed LD clumping for the IVs with the R package TwoSampleMR [2] v0.5.3 to identify independent pQTLs for each protein. We used a threshold of *r*^2^ < 0.001 to exclude dependent pQTLs in the local genetic regions as the default parameter implemented in the clump_data function.

### MR analyses using the TwoSampleMR R package

We used R package TwoSampleMR [2] v0.5.3, which includes two primary methods: For every single SNP, the most basic way, Wald ratio, was used; for multiple SNPs without pleiotropy, inverse variance weighted (IVW) estimator was used. This is the simplest way, and it is a meta-analysis of each Wald ratio for each SNP. The regression is constrained to pass through the origin, thus leading to a zero intercept. This package also implements the harmonization steps before performing MR, and these steps are listed here: (1) correct the wrong effect/non-effect alleles, (2) correct the strand issues, (3) fix the palindromic SNPs, and (4) remove the SNPs with incompatible alleles.

In our MR analysis, proteins from each tissue were set as the exposures and 211 complex human phenotypes as the outcomes (Fig. 1). As not all proteins within and between tissues nor all diseases are independent, we used a false discovery rate (FDR) < 0.05 as our multiple-test correction approach. The MR results were plotted as heatmaps using the geom_tile function from the R package ggplot2 [26]. Miami plots for the cis-only analysis using the geom_point function from the R package ggplot2 [26].

### Colocalization analyses on exposure with outcome

To remove the LD bias in MR analyses, we performed colocalization analysis using both coloc.abf function from R package coloc [3] v3.1 and coloc.susie function from R package coloc [27] v5.1 with a wrapper for susie_rss function from susieR [28] package v0.11.42. We first downloaded the full GWAS summary statistics for each disease/risk factor from the IEU OpenGWAS project [23] as VCF files [24]. We next set the window size to ± 500 kb centering on IV per protein-phenotype pair. We used the default priors, with p1 as 1 × 10^−4^, p2 as 1 × 10^−4^, and p12 as 1 × 10^−5^. Evidence for colocalization was assessed using the posterior probability (PP) for hypothesis 4 (indicating there is an association for both protein and disease and they are driven by the same causal variant(s)). We used PP.H4_final > 80% as a threshold to suggest that associations were highly colocalized. Under the assumption of only a single causal variant, if there were multiple instrumental variables used in MR, we calculated the average PP.H4 from the coloc.abf output. Under the assumption of multiple causal variants exist [28], we used the maximum PP.H4 of multiple credible sets from the coloc.susie output.

### Steiger filtering on inference direction from exposure to outcome

To mitigate the potential impact of reverse association (that is, the protein as outcome and phenotype as exposure), we used the Steiger filtering approach to identify the correct directions of inference (that is, the protein as exposure; phenotype as outcome). Only the protein-phenotype pairs with the correct direction were kept. Specifically, we used the directionality_test function implemented in R package TwoSampleMR [2] v0.5.3.

### Replication strategy for protein-phenotype associations

To replicate the protein-phenotype associations in this study, we used the full MR results from the previous plasma proteome-by-phenome-wide study [7]. Specifically, we extracted the full MR results on all protein-phenotype associations using the pqtl function implemented in R package epigraphdb [29] v0.2. Additionally, we used the significant result from a published study on brain proteome by seven neurological phenotypes [6]. The replication rate was calculated based on the number of replicated associations over the number of both replicated and not replicated associations.

### Cross-tissue comparisons of MR results

#### Direct protein-phenotype effect size comparisons of MR analyses across tissues

To compare the directions of effects across tissues given the same protein-phenotype pairs, we used the significant MR results (FDR < 0.05) with meta-analyzed genome-wide significant cis and trans pQTLs and the strong colocalization evidence (PP > 80%) across three tissues. We used rectangles (geom_tile function) to visualize each detailed cross-tissue MR estimate of the protein-phenotype association. We used scatterplots to visualize all available MR estimates between two tissues.

#### Phenotype-category proportion comparisons of MR analyses across tissues

To compare phenotype-category proportions of MR analyses across tissues, we used combined and split (cis-only and trans-additional) MR results by instrumental variables. Cis-only MR results were from protein-phenotype associations in common between workflow-a and workflow-b. Trans-additional MR results were from protein-phenotype associations unique to workflow-b alone. We used bar plots to visualize the proportions. We performed two-sided proportion tests for the overall phenotype-category proportions of MR analyses between each pair of three tissues.

#### Replication rates of the pQTL datasets between tissues

To calculate the replication rates of the pQTL datasets, we used Storey’s pi0 estimates [30] for deriving the replication rate (pi1 statistic) between the two tissue types. We calculated the replication rate of proteins used in MR between each tissue pair and found that the replicated rates were high (> 0.7) for all three pairwise comparisons (CSF vs plasma, pi1 = 0.97; CSF vs brain, pi1 = 0.77; plasma vs brain, pi1 = 0.79).

### Enrichment of proteome-wide MR with the druggable genome

Finan and colleagues [31] used targets of first-in-class drugs licensed since 2005; the targets of drugs currently in late-phase clinical development; information on the preclinical phase small molecules with protein binding measurements reported in the ChEMBL database [32]; as well as genes encoding secreted or plasma membrane proteins that form potential targets of monoclonal antibodies and other biotherapeutics. The authors identified 4479 genes as the latest druggable genome set and further classified these genes into three tiers: Tier 1 contained 1427 genes encoding targets of approved small molecules, biotherapeutic drugs, and clinical-phase drug candidates. Tier 2 included 682 genes encoding targets with known bioactive drug-like small-molecule binding partners and with more than 50% identity with drug targets. Tier 3 (3A and 3B) had 2370 genes encoding secreted or extracellular proteins, more distantly similar proteins to approved drug targets, plus proteins within key druggable gene families not already included in the first two tiers.

Altogether, we overlapped the 969 possible proteins passing QC with the 4479 genes (all three tiers) from the druggable genome using the Ensembl gene ID of the encoding genes.

We further assessed the overlap based on whether the protein was used in MR with cis or trans IVs and based on the druggable genome tiers. Similar to the previous study [7] using plasma pQTLs, we also calculated the enrichment of top pQTL MR findings with the druggable genome.

In CSF, we kept 144 protein-phenotype associations (80 proteins on 64 phenotypes) with both MR and colocalization evidence. We grouped the 80 proteins into four tiers based on their druggability: tier 1 contained 20 proteins, tier 2 contained 3 proteins, tier 3 contained 46 proteins, and tier 4 contained 11 proteins as unclassified. We classified 64 phenotypes into seven groups: 17 biological traits, 11 blood traits, 10 cancer types, one personality trait, two neurological diseases, 22 non-neurological diseases, and one other trait.

In the plasma, we used 96 protein-phenotype associations (52 proteins on 49 phenotypes) with both MR and colocalization evidence. We grouped the 52 proteins into four tiers based on their druggability: tier 1 contained 10 proteins, tier 2 contained nine proteins, tier 3 contained 24 proteins, and tier 4 contained nine proteins as unclassified. The 49 phenotypes were stratified into six groups: 14 biological traits, nine blood traits, four cancer types, one personality trait, two neurological diseases, and 19 non-neurological diseases.

In the brain, we analyzed 16 protein-phenotype associations (nine proteins on 15 phenotypes) with both MR and colocalization evidence. We grouped the nine proteins into four tiers based on their druggability: tier 1 did not contain any proteins, tier 2 contained two proteins, tier 3 contained four proteins, and tier 4 contained three proteins as unclassified. The 15 phenotypes were stratified into five groups: six biological traits, two blood traits, one cancer type, one neurological disease, and five non-neurological diseases.

The protein-phenotype associations with MR and colocalization evidence of each tissue were color-coded per their four corresponding druggability tiers.

### Drug repurposing

To obtain information on drug compounds that target proteins with pQTLs from this study, we used the DrugBank [33] database (as of 1/3/2020). This is a manually curated database that maintains profiles for > 15,000 drugs. For our analysis, we focused on the protein target for each drug compound. For each protein assayed, we identified all drugs in the DrugBank with a matching protein target based on UniProt ID [34]. We further integrated the MR and colocalization results on protein-phenotype associations into the overlap of proteins as drug targets. Additionally, we added the indication information (“drug indications”) or side-effect (“drug warnings”) using the ChEMBL [32] database (as of 11/15/2021) for these drug compounds.

## Results

### Inferring multi-tissue protein effects on disease-related phenotypes using cis study-wide significant pQTLs as IVs

#### Washington University cohort-specific analyses

First, we performed analyses using only the pQTLs identified on the WashU cohort that included 835 CSF, 529 plasma, and 380 brain samples in which 713, 931, 1079 proteins were measured using the SOMAscan platform (1305 panel) and passed QC in each tissue, respectively.

**Fig. 2:**
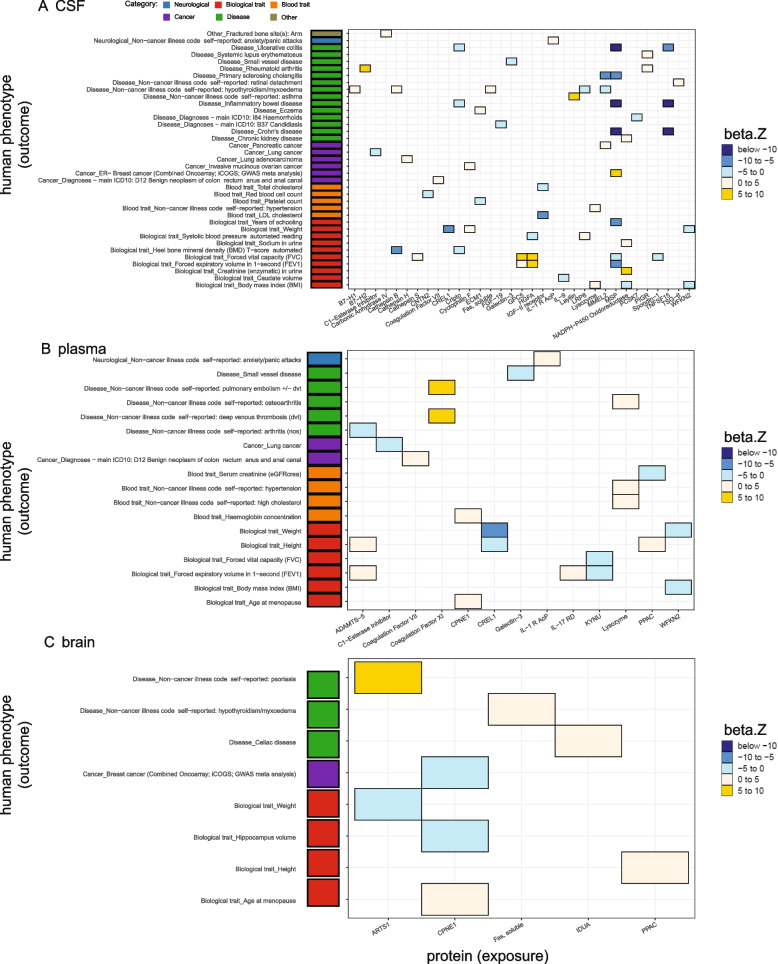
Significant protein-phenotype associations identified using *cis*-only study-wide pQTLs as instrumental variables. Heatmaps were generated using the analyses on the WashU cohort only. **A** Thirty-three proteins against 37 diseases in CSF. **B** Thirteen proteins against 18 diseases in the plasma. **C** Five proteins against eight diseases in the brain. Colors were coded by 5 bins after cutting *z*-normalized beta MR estimate: below − 10 as dark blue, − 10 to − 5 as dodger blue, − 5 to 0 as cadet blue1, 0 to 5 as antique white1, and 5 to 10 as gold. Phenotype categories were listed on the left side as a bar plot (neurological diseases as blue, biological traits as red, blood traits as orange, cancers as purple, non-neurological diseases as green, and other risk factors as khaki)

**Fig. 3:**
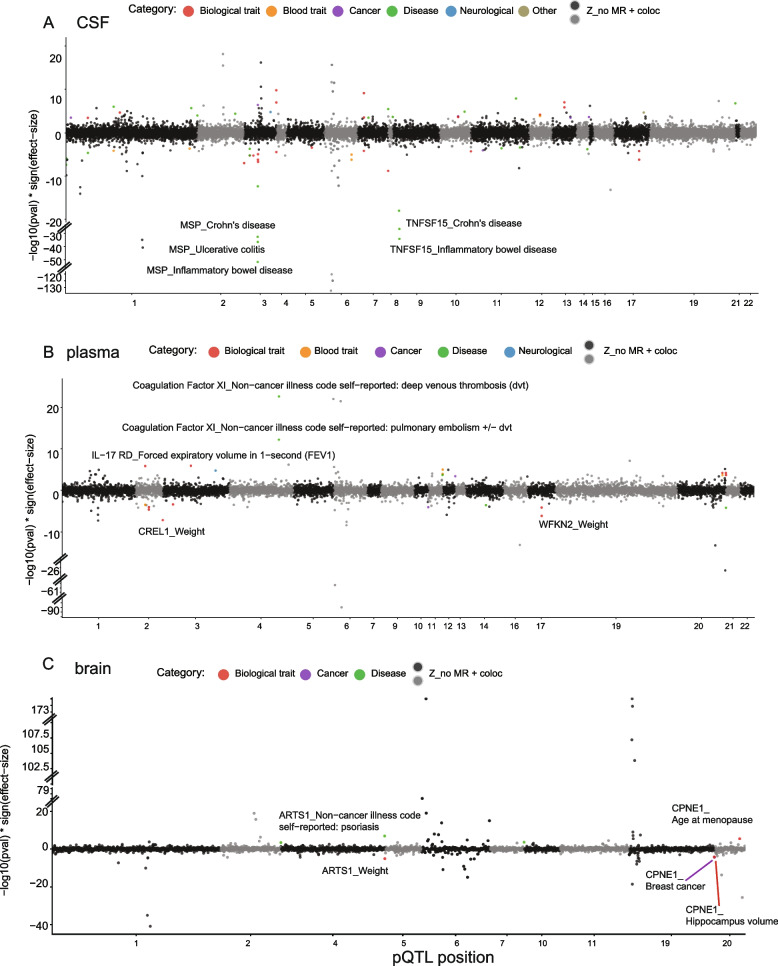
Miami plots for the *cis*-only study-wide pQTLs as IVs for all MR and colocalization analyses. Each dot represents the MR results for proteins on human phenotypes. **A** CSF. **B** Plasma. **C** Brain. Phenotype categories were color-coded: biological traits as red, blood traits as orange, cancers as purple, non-neurological diseases as green, neurological diseases as blue, and other risk factors as khaki; for protein-phenotype associations not significant or not colocalized, the color is dark/light gray

We initially performed the MR analyses including the study-wide significant cis pQTLs (103, 47, 15 protein-locus pairs in the CSF, plasma, and brain, respectively). Horizontal pleiotropy can lead to false-positive results in MR analyses. Although it is known that cis pQTLs are less likely to be susceptible to horizontal pleiotropy than trans pQTLs [1, 7, 9], we removed pleiotropic cis pQTLs (defined as associated with five or more proteins) as IVs. We also performed colocalization analyses to examine the confounding effect of LD. Colocalization can provide complementary supporting evidence of inference by decreasing the likelihood of confounding by LD. Furthermore, we used the Steiger filtering to identify the correct directions of inference. We kept only protein-phenotype pairs that protein has effects on phenotype thereafter (Figs. 2 and 3, Table 1, Additional file 2: Tables S2-S4).

**Table 1:** Summary of replication on MR results using study-wide *cis* pQTLs with WashU cohort

Tissue	Total	Replicated	Not replicated	Novel
CSF	60	21	12	27
Plasma	23	20	2	1
Brain	8	6	1	1

Replicated: *p*-value < 0.05 and the same direction of effect size in the prior plasma study by Zheng et al.’s 2020 full MR results; not-replicated: *p*-value ≥ 0.05 and/or the opposite direction of effect size in the prior plasma study; novel: protein-phenotype pairs not found in the prior plasma study

We found that 33 CSF proteins were associated with 37 phenotypes, (Figs. 2A and 3A) with both significant MR results (FDR < 0.05) and strong colocalization evidence (PP > 80%), 13 plasma proteins were associated with 18 phenotypes (Figs. 2B and 3B), and five brain proteins were associated with eight phenotypes (Figs. 2C and 3C). In CSF (Fig. 2A), two proteins were associated with multiple phenotypes from the same category: (1) MSP was negatively associated with four general diseases (primary sclerosing cholangitis, Crohn’s disease, inflammatory bowel disease (IBD), ulcerative colitis (UC)) and three biological traits (years of schooling, forced vital capacity (FVC), forced expiratory volume in 1-second (FEV1)) and (2) TNFSF15 was negatively associated with Crohn’s disease, IBD, and UC.

In the plasma (Fig. 2B), six proteins were associated with multiple phenotypes from the same category: (1) ADAMTS-5 was positively associated with two biological traits (height and FEV1), (2) coagulation factor XI was positively associated with two diseases (deep venous thrombosis (DVT) and pulmonary embolism ± DVT), (3) CREL1 was negatively associated with two biological traits (height and weight), (4) KYMU was negatively associated with two biological traits (FVC and FEV1), (5) lysozyme was positively associated with two blood traits (hypertension and high cholesterol), and (d6) WFKN2 was negatively related to two biological traits (body mass index (BMI) and weight).

In the brain (Fig. 2C), protein CPNE1 was associated with multiple biological traits: it had a positive association with age at menopause and a negative association with hippocampus volume.

Among all these protein-phenotype pairs, we replicated previously reported findings in the plasma [7, 29] and brain [6] (Table 1, Additional file 2: Tables S2, S3). The replication rates in the CSF, plasma, and brain were 64%, 91%, and 86%, respectively, when compared with the plasma studies [7, 29]. On the other hand, our results did not replicate three previous findings in the brain after overlapping both proteins and phenotypes: CPNE1 on intelligence, cathepsin H on AD, and ALT on intelligence, where the study used a brain pQTL dataset from 144 samples [6].

Meanwhile, we uncovered 45%, 4%, and 12% novel protein-phenotype associations, in the CSF, plasma, and brain, respectively (Table 1, Additional file 2: Table S4).

#### pQTL meta-analyses uncovered additional protein-phenotype pairs

Furthermore, to increase the power of our analyses, we performed two-sample MR using summary statistics from meta-analyses for CSF and plasma independently (Fig. 1B). CSF meta-analysis included two cohorts including PPMI released in 2019 (*N* = 132) [20] and WashU [16]. We included 709 CSF proteins shared in both studies.

For the plasma, we leveraged two cohorts including INTERVAL (*N* = 3301) [21] and SCALLOP (*N* = 30,931) [22], that were meta-analyses with WashU [16]. For WashU and INTERVAL, we included 746 plasma proteins shared in both studies, and for WashU, INTERVAL and SCALLOP, we included 49 plasma proteins shared in three studies.

These meta-analyses yielded 31 additional CSF and 215 plasma pQTLs, which led to 10 additional CSF proteins associated with 13 phenotypes (Additional file 1: Fig. S3A, Additional file 2: Tables S5-S7) with significant MR results and strong colocalization evidence. Our analyses also identified 12 additional plasma proteins associated with 14 phenotypes (Additional file 1: Fig. S3B, Additional file 2: Tables S5-S7). In CSF (Additional file 1: Fig. S3A), protein IL1 receptor-type1 (IL-1 sRI) was associated with multiple phenotypes: it was negatively associated with three general diseases (IBD, Crohn’s disease, UC) while positively correlated with asthma. In the plasma (Additional file 1: Fig. S3B), the protein haptoglobin was negatively associated with two blood traits (LDL and total cholesterol) while positively related to height. No protein-phenotype pairs had an opposite effect size before and after meta-analysis (36 in CSF and four in plasma).

We successfully replicated the finding that CSF IL-1 sRI increased the risk of asthma, which was not found in our initial analyses (as meta-analyses increased the statistical power of IL-1 sRI pQTL *p*-value from 1.09 × 10^−18^ to 2.32 × 10^−25^). IL-1 receptor antagonist has been tested to attenuate asthmatic symptoms in animal models [35]. We also replicated the finding on plasma haptoglobin associated with reduced LDL and total cholesterol levels, as reported by Boettger and colleagues [36]. Moreover, we highlighted the risky effects of plasma B7-H2 (or ICOS ligand) on rheumatoid arthritis (RA), as it has been validated in a mouse model of RA that anti-ICOS ligand domains can help reduce the disease symptoms [37].

### Inferring multi-tissue protein effects on disease-related phenotypes using both cis and trans genome-wide significant pQTLs as IVs

In the previous section, we only used cis pQTLs that passed the most stringent study-wide threshold. This threshold, however, may miss the real biological signals. Therefore, a more permissive threshold could reveal additional signals. To increase the power of MR analyses, we expanded our MR analyses by including potentially non-pleiotropic cis and trans pQTLs as instrumental variables that passed the genome-wide threshold (*p* < 5 × 10^−8^, *F* ≥ 10, and associated with fewer than 5 proteins).

#### Washington University cohort-specific analyses

**Fig. 4:**
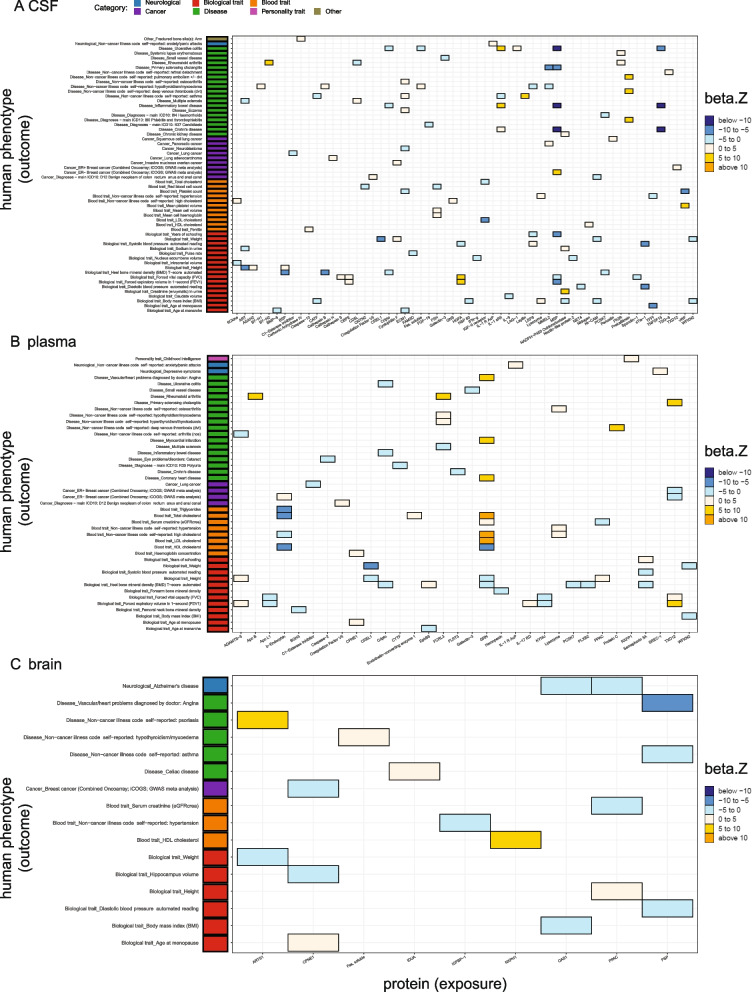
Significant protein-phenotype associations identified using *cis* and *trans* genome-wide pQTLs as instrumental variables. Heatmaps were generated using the analyses on the WashU cohort only. **A** Fifty-eight proteins against 58 diseases in CSF. **B** Thirty-two proteins against 44 diseases in the plasma. **C** Nine proteins against 16 diseases in the brain. Colors were coded by 6 bins after cutting *z*-normalized beta MR estimate: below − 10 as dark blue, − 10 to − 5 as dodger blue, − 5 to 0 as cadet blue1, 0 to 5 as antique white1, 5 to 10 as gold, and above 10 as orange. Phenotype categories were listed on the left side as a bar plot (biological traits as red, blood traits as orange, cancers as purple, non-neurological diseases as green, neurological diseases as blue, personality traits as pink, and other risk factors as khaki)

With this new threshold, 169, 116, 50 cis and trans pQTLs in the CSF, plasma, and brain, respectively, were used for MR and colocalization analyses for the WashU cohort analyses (Fig. 4, Table 2, Additional file 2: Tables S8-S10). This led to the identifications of 58 CSF proteins associated with 58 phenotypes (Fig. 4A), 32 plasma proteins on 44 phenotypes (Fig. 4B), and nine brain proteins on 16 phenotypes (Fig. 4C) with significant MR results (FDR < 0.05) and strong colocalization evidence (PP > 80%).

**Table 2:** Summary of replication on MR results using genome-wide *cis* and *trans* pQTLs with WashU cohort

Tissue	Total	Replicated	Not replicated	Novel
**CSF**	110	37	25	48
**Plasma**	67	37	18	12
**Brain**	17	10	5	2

Replicated: *p*-value < 0.05 and the same direction of effect size in the prior plasma study by Zheng et al.’s 2020 full MR results; not-replicated: *p*-value ≥ 0.05 and/or the opposite direction of effect size in the prior plasma study; novel: protein-phenotype pairs not found in the prior plasma study

Similar to the cis-only-pQTL analyses above, we replicated findings reported previously from a plasma [7, 29] and a brain study [6] (Table 2, Additional file 2: Table S8). In these new analyses, there were a total of 37, 37, and 10 CSF, plasma, and brain protein-phenotype pairs that were previously reported, respectively. Several protein-phenotype associations, however, did not replicate due to the weaker instrumental variables compared to the prior studies (Table 2, Additional file 2: Table S9). Additional novel protein-phenotype findings (48, 12, two in CSF, plasma, and brain, respectively) were also revealed after including both cis and trans genome-wide significant pQTLs (Table 2, Additional file 2: Table S10).

#### pQTL meta-analyses identified additional protein-phenotype pairs

We performed meta-analyses from two cohorts of CSF and three cohorts of plasma, leading to additional 313 CSF and 711 plasma pQTLs as IVs. This approach identified 21 additional CSF proteins associated with 17 phenotypes (Additional file 1: Fig. S4A, Additional file 2: Tables S11-S13), and 15 plasma proteins were associated with 15 phenotypes (Additional file 1: Fig. S4B, Additional file 2: Tables S11-S13). No protein-phenotype pairs had an opposite effect size before and after meta-analysis (70 in CSF and three in plasma).

**Fig. 5:**
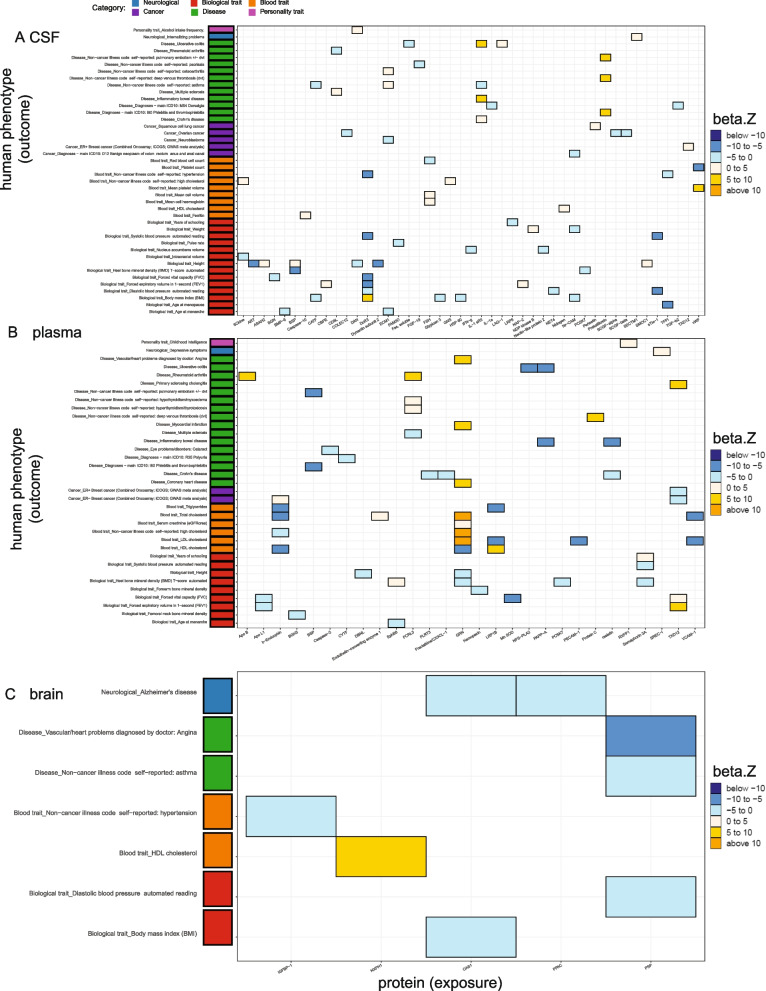
Additional significant protein-phenotype associations were identified after including *cis* and *trans* genome-wide pQTLs as instrumental variables. Heatmaps were generated using the analyses after meta-analyses. **A** Forty-five proteins against 42 diseases in CSF. **B** Twenty-eight proteins against 35 diseases in the plasma. **C** Five proteins against seven diseases in the brain. Colors were coded by 6 bins after cutting *z*-normalized beta MR estimate: below − 10 as dark blue, − 10 to − 5 as dodger blue, − 5 to 0 as cadet blue1, 0 to 5 as antique white1, 5 to 10 as gold, and above 10 as orange. Phenotype categories were listed on the left side as a bar plot (biological traits as red, blood traits as orange, cancers as purple, non-neurological diseases as green, neurological diseases as blue, and personality traits as pink)

To identify what was absent in our initial analyses that included cis-pQTLs, we compared two results from the study-wide versus the genome-wide *p*-value thresholds (Fig. 5, Additional file 2: Table S14). We identified additional associations for 45 CSF proteins with 42 phenotypes (Fig. 5A), 28 plasma proteins with 35 phenotypes (Fig. 5B), and five brain proteins with seven phenotypes (Fig. 5C).

In CSF (Fig. 5A), three proteins (DcR3, IL-1 sRII, Prekallikrein) were associated with more than two phenotypes within each category: (1) DcR3 was negatively associated with four biological traits (FVC, FEV1, diastolic blood pressure (DBP), systolic blood pressure (SBP)) and hypertension, while positively associated with BMI. (2) IL-1 sRII was positively associated with three diseases (Crohn’s disease, IBD, UC), while negatively associated with asthma. (3) Prekallikrein was positively associated with three diseases (Phlebitis and thrombophlebitis, DVT, and pulmonary embolism ± DVT).

In the plasma (Fig. 5B), two proteins (b-Endorphin, GRN) were associated with multi-phenotypes within each category: (1) b-Endorphin was negatively connected to four blood traits (HDL, total, high cholesterol, triglycerides) while positively associated with ER-negative breast cancer, and (2) GRN was positively related to four blood traits (serum creatinine, LDL, total, high cholesterol) and negatively related to HDL cholesterol. GRN was also found in positive associations with three cardiovascular diseases (coronary heart disease (CHD), myocardial infarction, and angina) and negative associations with two biological traits (heel bone mineral density (BMD) and height).

In the brain (Fig. 5C), two proteins (PSP and OAS1) were concordantly associated with more than two phenotypes: (1) PSP was negatively connected to two general diseases (angina and asthma) and one biological trait (DBP), and (2) OAS1 was negatively related to one biological trait (BMI) and one neurological disease (AD). Particularly, we showed a consistent finding that the brain OAS1 is protective against AD risk as recently published by Magusali and colleagues [38]. Magusali et al. [38] reported that OAS1 is required to limit the pro-inflammatory response of human induced pluripotent stem cell-derived microglia.

### Cross-tissue comparisons on tissue consistency of the protein-phenotype effects

**Fig. 6:**
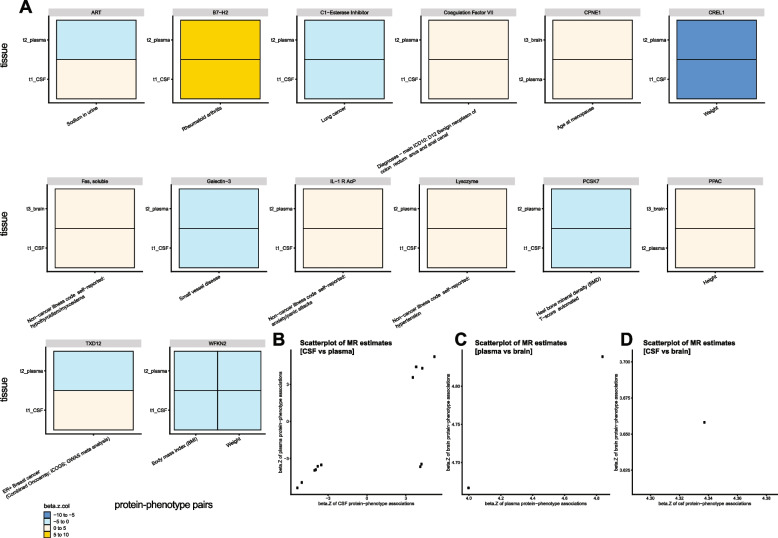
Cross-tissue MR estimate comparisons. **A** Heatmaps were generated on the MR estimates given the same protein-phenotype pairs with a PP > 80% when performing colocalization. Colors were coded by 4 bins after cutting *z*-normalized beta MR estimate: − 10 to − 5 as dodger blue, − 5 to 0 as cadet blue1, 0 to 5 as antique white1, and 5 to 10 as gold. **B** Scatter plot of CSF vs plasma MR estimates on the same protein-phenotype associations. **C** Scatter plot of plasma vs brain MR estimates on the same protein-phenotype associations. **D** Scatter plot of CSF vs brain MR estimates on the same protein-phenotype associations

To investigate whether the directions of effects were consistent across tissues given the same protein-phenotype pairs, we compared the significant MR results (FDR < 0.05) using meta-analyzed genome-wide significant cis and trans pQTLs with strong colocalization evidence (PP > 80%) across three tissues. We identified 15 pairs in more than one tissue, in which 13 pairs had consistent MR estimates (Fig. 6). Among these 13 tissue-consistent pairs, 10 were concordant between CSF and plasma (Fig. 6A, B), two between plasma and brain (Fig. 6A, C), and one between CSF and brain (Fig. 6A, D). For example, WFKN2 levels from CSF and plasma were consistently associated with two phenotypes: BMI and weight (Fig. 6A).

Two pairs showed discordant MR effect sizes (Fig. 6A, B): (i) higher CSF ART (or AGRP, Agouti-related protein) was associated with higher levels of sodium in the urine, whereas higher plasma ART was associated with lower levels of the same trait; (ii) higher CSF TXD12 was associated with a higher risk of the ER-positive Breast cancer, whereas higher plasma TXD12 was associated with lower risk of the same phenotype. Overall, we found a small proportion of tissue-dependent protein effects on certain phenotypes.

**Fig. 7:**
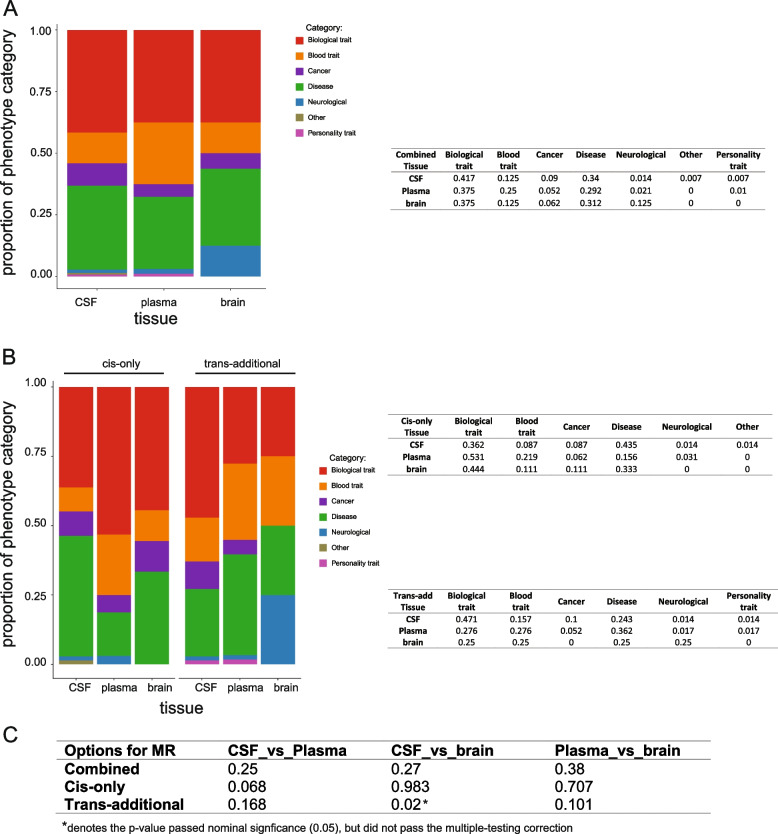
Phenotype-category proportions of MR analyses from each tissue. Barplots were used to visualize the proportions of phenotype category per tissue and the percentage of each proportion was listed in the table in parallel. The MR results are from **A** combined analyses. **B** After splitting into *cis*-only and *trans*-additional findings by instrumental variables used. **C** Table summarizing the *p*-value of the proportion test (two-sided) for the overall phenotype-category proportions of MR analyses between each pair of three tissues. Phenotype categories were color coded as biological traits as red, blood traits as orange, cancers as purple, non-neurological diseases as green, neurological diseases as blue, personality traits as pink, and other risk factors as khaki

To estimate the enrichment of phenotypes in different tissues, we compared phenotype-category proportions of MR analyses from each tissue (Fig. 7). Even plasma protein MR findings showed a higher proportion of blood traits, and brain protein MR results presented a higher proportion of neurological diseases, we found no statistically significant proportions on phenotype category across tissues (Fig. 7A, C). As our previous study [16] suggested that trans, but not cis, pQTLs may be tissue-specific, we further split the disease category of MR analyses from each tissue into cis-only and trans-additional findings to determine if there is any tissue-specific phenotypic enrichment (Fig. 7B, C). We found the pairwise tissue comparisons involved in the brains on proportions of disease category using IVs from cis-only analyses had a larger *p*-value than from trans-additional analyses from the proportion test. This observation may be underpowered but can be partially explained by our prior findings [16] that trans-pQTLs tend to be more tissue-specific than cis-pQTLs.

To determine whether the phenotype enrichment was affected by the statistical power across tissues because of the differences in sample size and protein, we first calculated the statistical power for pQTL identification in each tissue, and we next did sensitivity analyses by keeping only the same protein sets for the estimation of the disease enrichment. Our current study was well-powered (Additional file 1: Fig. S1) for cis-pQTL-based MR analyses given the same protein available across all tissues. However, for trans-pQTL-based MR analyses, the statistical power for CSF and brain were below 0.8 (Additional file 1: Fig. S1), indicating that we were underpowered to detect trans-pQTLs under the assumption that the protein should have at least one trans-pQTL in all tissues. Moreover, this assumption will be too stringent as we cannot ensure proteins from different tissues measured by the same platform share the same genetic structure. Thus, the phenotype enrichment analyses using trans-pQTL-based MR results may be underpowered to provide robust estimates.

Even if we used the same panel (SOMAscan 1305 panel) in all three tissues, different subsets of proteins passed QC in each tissue could bias the downstream disease enrichment analysis. To correct this bias, we performed the enrichment analysis using only the 411 proteins that passed QC in all three tissues (Additional file 1: Fig. S5). Even the proportion of neurological diseases was higher in the brain than in the plasma (brain: 10%; plasma: 3.3%) and the proportion of blood traits was higher in plasma than in the other two tissues (plasma: 23%; CSF: 7.8%; brain: 10%), these differences were not statistically different (CSF vs plasma *p*-value = 0.2; plasma vs brain *p*-value = 0.952; CSF vs brain *p*-value = 0.780). This is consistent with the current MR results including all proteins that passed QC in any tissues, indicating that the phenotype enrichments are not biased by different protein sets.

### Overlap proteins with druggable genome

Moreover, we overlapped our proteins having strong MR and colocalization evidence with the druggable genome reported by Finan and colleagues [31]. To assess the overlap of the proteins identified in our MR analyses and based on the druggable genome tiers, we performed an enrichment analysis as described before [7] (Additional file 2: Table S15). Of the proteins associated with the studied phenotypes, 86.3% (69/80), 82.7% (43/52), and 66.7% (6/9) proteins in CSF (Additional file 1: Fig. S6A), plasma (Additional file 1: Fig. S6B), and brain (Additional file 1: Fig. S6C), respectively, intersected with the first three druggable genome tiers. These overlapping proteins were associated with seven, six, and four unique phenotypic categories (the “Methods” section).

### Drug repurposing

**Fig. 8:**
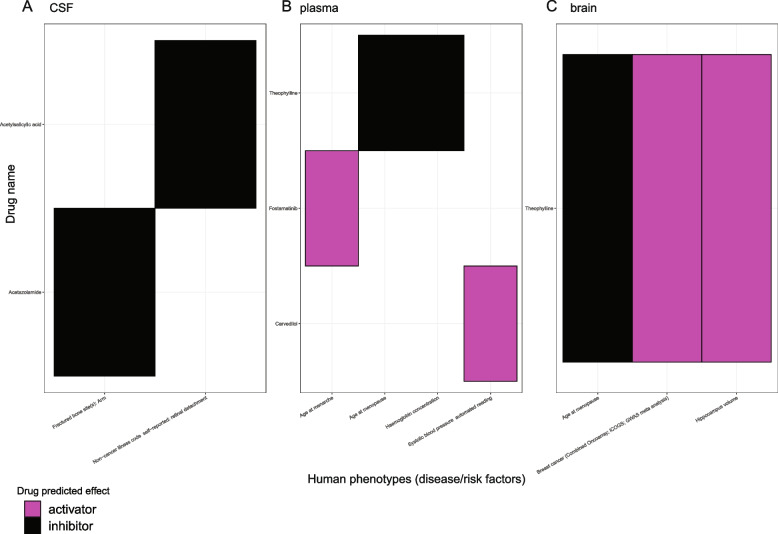
Phenotype-drug pairs after integration of protein-phenotype associations from MR and drug-protein interactions from DrugBank & ChEMBL databases. Heatmaps were used to visualize drug-name against phenotype for the drug target repurposing purpose. The drug-predicted effects were listed as follows: **A** in CSF, two drugs can be used as an inhibitor given a positive estimate from MR analyses; **B** two activators and two inhibitors in plasma; and **C** two activators and one inhibitor in brain. Colors were coded: activator as magenta and inhibitor as black

Finally, to repurpose the known drug compounds for the phenotypes, we linked the inference results using meta-analyzed genome-wide significant cis and trans pQTLs with two drug databases. Using the DrugBank database [33] to first assign protein targets with a compound, which is curated by UniProt [34] and the ChEMBL database [32] to further keep the maximum clinical trial phase as “4” from the indication information and no side-effects, we identified two, three, one protein in CSF, plasma, and brain, respectively, connected with at least one compound for one disease-related phenotype (Fig. 8, Additional file 1: Fig. S7, S8, Additional file 2: Table S16). For CSF proteins as targets (Fig. 8A), two drugs can be used as an inhibitor given a positive estimate from MR analyses; for proteins from the plasma (Fig. 8B) and brain (Fig. 8C), two and two drugs, respectively, were predicted as activators, whereas two and one, respectively, were inferred as inhibitors. For example, plasma N-terminal pro-BNP can be targeted by carvedilol to lower the SBP. CSF TSG-6 can be targeted by acetylsalicylic acid in treating retinal detachment. Brain CPNE1 was found as a target of a small molecule drug, called theophylline, and potentially regulates the size of hippocampus volume and age at menopause.

### Discussion

Here, our study revealed that 80 CSF, 52 plasma, and nine brain proteins were associated with 64, 49, and 15 human disease-related phenotypes, respectively. Of these, we identified 45.8%, 30.2%, and 12.5% novel protein-phenotype pairs in CSF, plasma, and brain, respectively. After integrating the published druggable genome results, we found that 66.7 to 86.3% of proteins, depending on tissues, could be potential therapeutic targets for a complex trait/phenotype. These results systematically tested the potential effects of proteins, as potential drug targets, on human diseases or risk factors by both MR and colocalization in a tissue-specific manner.

Our study is the first analysis that systematically evaluated the cross-tissue protein effects on over 200 phenotypes using pQTLs from three tissues. Our result can be used as a complementary resource to the plasma proteome-by-phenome-wide MR studies [7, 14]. Our current study generated a multi-tissue MR atlas, and thus, we did not pre-select the priori “tissue-specific” phenotypes. This strategy would be extremely helpful in the downstream comparisons of cross-tissue MR effects given the same protein-phenotype associations.

From the MR results using genome-wide significant pQTLs, we found 48, 12, and two novel protein-phenotype pairs in CSF, plasma, and brain, respectively (Table 2), which were absent from previous studies [6, 7]. We found the largest set of novel protein-by-phenotype associations was from CSF proteins, and it could be explained as this study used the largest CSF pQTL dataset at the time of the analyses. Our MR results revealed that plasma proteins, as well as CSF and brain proteins, can be prioritized in the disease pathogenesis and further used as druggable targets. Our study expanded the scale of inferring CSF and brain protein effects on diseases to the phenome-wide scale compared to prior protein-disease MR studies in CSF [11] and brain [6]. Our results highlight proteins with potential opportunities for developing treatment with clinical trials; however, further functional experiments, in vitro and in vivo, would be essential to validate these findings. We think additional preclinical and clinical studies are relevant as 67% of the FDA-approved drugs last year (2021) had strong genetic and genomic support [39]. We defined that protein-phenotype associations are consistent across tissues as tissue-shared effects, whereas protein-phenotype associations are opposite across tissues as tissue-specific effects. It would be easier to develop a drug for proteins with tissue-shared effects, as that is not depending on tissue types.

We found 15 protein-disease pairs that were found in more than one tissue, and 87% (13 out of 15) of the shared protein effects on phenotypes were consistent across different tissues. We found two proteins with opposite effects across tissues. No previous studies have reported the two proteins with opposite associations between CSF and plasma: (1) ART protein level on the trait of sodium in urine level and (2) TXD12 on ER-positive breast cancer risk. It is important to note that these opposite effects were driven by different IVs used in each tissue as different sentinel pQTLs were found in those tissues. Opposite QTL effect sizes across tissues have been reported before, mainly for the expression QTL (eQTL). Fu and colleagues [40] reported that 4.4% eQTLs had opposite directions using blood and non-blood tissues. Mizuno and Okada [41] later performed a study on the opposite eQTL effects with more tissue types (48 tissues) from Genotype-Tissue Expression (GTEx) project (release-version-7). This later study highlighted that the opposite eQTL effects can be observed between closely related tissues such as different brain regions (for example, cerebellum versus brain cortex) and be estimated as 7.4% of the eQTL-genes. These two studies pinpointed that these opposite genetic effects on gene expression between tissues can further contribute to the development of complex traits in a tissue-dependent manner. Another MR study [42] (using eQTLs as IVs) on phenome-wide (395 complex traits) reported tissue-dependent effects on the same phenotypes. In this study, we extended these observations from gene expression to the protein level.

Our MR and colocalization results were largely driven by cis-pQTLs. As cis-pQTLs are more sharable across tissues compared to trans-pQTLs, our results tend to be tissue-shared. As for estimating the enrichment of diseases in different tissues, we did not find any tissue-specific disease enrichment. This can be explained because the pQTLs used in MR and colocalization were shared among three tissues or because disease processes may implicate more tissues than initially expected.

The fact that we did not find any clear enrichment of phenotype category by tissue (for example, neurological diseases in the brain and/or CSF) but found general protein-phenotype associations in those analyses may provide new avenues to understand these complex traits, and indicate that it may be necessary to investigate not only the primary disease-relevant tissue. For example, protein levels in CSF were associated with several autoimmune diseases (e.g., Crohn’s disease, UC, IBD), suggesting that these diseases may also have a brain component. For Crohn’s disease, multiple neurological effects have been reported [43], including myelopathy, posterior reversible encephalopathy syndrome, and chronic inflammatory demyelinating polyneuropathy. For UC, it was found in patients with peripheral nerve disorders and cerebrovascular disorders [44]. As IBD is comprised of Crohn’s disease and UC, these neurological effects apply to IBD in general [45]. Therefore, CSF proteins can play roles in the pathogenesis of these autoimmune diseases. Proteins from the plasma were associated with multiple none-blood traits (e.g., cancers: D12 benign neoplasm of colon rectum anus and anal canal or ER− Breast cancer or ER+ breast cancer or lung cancer; neurological-traits: depressive symptoms/anxiety; personality: childhood intelligence). For cancers, this can be explained by the cancer cells spreading to other organs (colon, breast, or lung) via the blood [46]. For neurological or personality traits, proteins in the plasma can be functioning in the brain indirectly via the blood-brain barrier [47]. Proteins from the brain were associated with multiple none-CNS traits (e.g., breast cancer; celiac disease, angina). For breast cancer, proteins in the brain can function in blood indirectly via the blood-brain barrier. For celiac disease, it was reported to have multiple neurological and psychiatric effects [48]. For angina, it was observed to be associated with mental stress-induced inferior brain activation [49]. Therefore, brain proteins can play roles in the pathogenesis of these non-CNS diseases.

This study is the first time the strategy without selecting a priori “tissue-specific” phenotypes was applied to multi-tissue pQTLs; similar strategies have been used in multi-tissue eQTL studies previously. GTEx consortium in 2020 [50] used gene expression QTLs from 49 tissues of v8-release to analyze the role of these eQTLs in genetic associations for 87 human traits (including asthma, multiple sclerosis, Parkinson’s diseases, Crohn’s diseases, high cholesterol, etc.). The authors from the GTEx consortium analyzed all pairwise combinations of 87 phenotypes and 49 tissues without pre-selecting tissue-relevant phenotypes, e.g., brain tissues were not just used for the CNS traits; whole blood was not just for the blood traits. The same strategy was also originally published in 2018 [51]. The authors inferred tissue enrichment of the eQTL from GTEx v6p-release in 18 complex traits. The authors reported most enriched tissues per trait also existed in less biologically obvious tissues, for example, eQTLs in the ovary were enriched in coronary artery disease; eQTLs in the skin were enriched in Alzheimer disease. This indicated two possibilities interpreted by Gamazon and colleagues [51]: (a) shared regulation with the actual tissues of action or (b) new pathogenic tissues.

The finding of protein-phenotype links in the tissue that was not initially expected is not the only highlight in this study which can point to new pathogenic events. In the case of cancer, having a link between cancer and brain/CSF may just be explained as the protein implicated in cancer via an indirect pathway. This pathway may contain many factors from the brain/CSF proteins (as the starting point) to the cancer of certain organs (as the ending point), and it is the meaning of vertical pleiotropy, but not horizontal pleiotropy, thus it does not violate the assumption of MR, but indicates that this complex biology of the human body and these traits. In addition, finding the same protein associated with multiple phenotypes point to the shared pathogenic processes etiologies. We found 34, 22, and 5 proteins from CSF, plasma, and brain, respectively, were associated with multiple phenotypes (Additional file 1: Fig. S8). This is because IVs for the same protein were shared across multiple phenotypes. For example, CSF MSP was associated with eight phenotypes in total. The genetic correlations between each phenotype (Additional file 1: Fig. S8A) revealed that not all eight phenotypes are highly correlated with each other, though some phenotypes were indeed highly correlated, such as primary sclerosing cholangitis (ieu-a-1112), Crohn’s disease (ieu-a-12), IBD (ieu-a-294), and UC (ieu-a-970). Four of these diseases (primary sclerosing cholangitis, Crohn’s disease, IBD, and UC) were reported to present comorbidities [52]. As IBD contains Crohn’s disease and UC, the comorbid patients with both IBD and primary sclerosing cholangitis can be further broken down into 80% of UC and 20% CD. From the previous genetic studies, this comorbidity may be formed from participants with a predisposition to autoimmune biliary injury via colonic inflammation. This indicated CSF MSP played an important role in multiple diseases sharing the same mechanisms for disease pathogenesis. Similar observations were held for plasma GRN (Additional file 1: Fig. S8B) and brain CPNE1 (Additional file 1: Fig. S8C).

Our study has several limitations. First, our bulk-tissue proteomic profiling was not cell type-specific. Thus, our estimation of protein-phenotype would be biased when using cell type-specific proteomic datasets if available. However, we were accounting for different tissues compared to the prior single-tissue studies [6, 7]. Second, the epitope-binding effect instead of true abundance change from aptamer-binding assay would create artifacts of pQTLs. Third, the pleiotropic IVs may still be missing due to our limited coverage of the whole human proteome. We and others are not able to detect horizontal pleiotropy due to the relatively small number of proteins measured in our current datasets. We were unable to use MR-Egger [53] or MR-PRESSO [54] to correct for horizontal pleiotropy as most of the proteins were only associated with one valid instrumental variable (*F* ≥ 10) after clumping. We were unable to distinguish whether the inferred causal relationships were truly causal due to failing to test for horizontal pleiotropy. Thus, our observations on protein-phenotype associations are consistent with a causal role or horizontal pleiotropy. Fourth, sensitivity analyses would not be possible for most QTL studies, as stated by Baird and colleagues [55]. As an alternative approach, Burgess and colleagues claimed as a guideline [1] that colocalization can help evaluate exposures such as proteins and gene expression, particularly when the MR result is derived from a single-gene region. Fifth, our pQTL dataset pre-meta-analysis is from an aging cohort. Even though we did not identify age-specific pQTLs in our dataset (age range from 37 to 107) [16], it may be possible that age-specific pQTLs exist for other age ranges, or that our dataset does not provide enough statistical power for the age range of this study; thus this could bias the results due to survival bias. Sixth, our brain pQTL study is from a single center. This reflected that post-mortem brain tissues are challenging to collect, and future genome-wide brain pQTL studies can be used for meta-analysis. Seventh, we were underpowered to detect more tissue-specific protein effects on the same phenotypes. We learned empirically that the power of identifying trans-pQTLs (Additional file 1: Fig. S1) for CSF and brain were below 0.8, whereas the power in plasma is 0.91, given the current sample size and assuming a protein is warranted with a trans-pQTL. As the sample size in each tissue is different, therefore the power to identify cis and trans pQTLs is different in each tissue, which can confound the results. The sample sizes of CSF and brain in this study are much smaller than the published plasma-based studies, thus may bias the cross-tissue findings. Eighth, our pQTL dataset pre meta-analysis was generated with a cohort mixed with AD cases and controls; our results may be confounded by the disease status, though we did not identify any disease-specific pQTLs [16].

### Conclusions

In summary, our work evaluates multi-tissue protein effects on disease-related phenotypes at a large proteome-by-phenome-wide scale. We prioritize six CSF proteins including MSP, TNFSF15, IL-1 sRI, DcR3, IL-1 sRII, and Prekallikrein; nine plasma proteins including ADAMTS-5, coagulation factor XI, CREL1, KYMU, lysozyme, WFKN2, haptoglobin, b-Endorphin, and GRN; and three brain proteins including CPNE1, PSP, and OAS1, as they were top ranked by the number of multiple phenotypes. We anticipate that in the future, much larger-scale studies using additional proteins from a more extensive set of tissues with more phenotypes will facilitate the drug repositioning process.

## Supplementary Information

**Additional file 1.** Supplementary figures (Fig. S1–S8).

**Additional file 2.** Supplementary tables (Table S1–S16).

## Data Availability

The accession numbers for each protein with pQTL set (at a threshold of 1e-5) from all tissues are available at the GWAS-catalog (https://www.ebi.ac.uk/gwas/studies/) [56]: ranging from GCST90225560 to GCST90225998 and these will contain all significant associations at a threshold of 1e-5, along with the appropriate metadata. The results of all analyses based on the multi-tissue pQTLs are included in this published article and its supplementary information files. The same multi-tissue pQTL summary statistics can also be accessed at https://www.niagads.org/datasets/ng00102. Other web resources used in this article are listed below: IEU OpenGWAS project website, https://gwas.mrcieu.ac.uk/ DrugBank link to UniProt ID, https://www.uniprot.org/database/DB-0019 ChEMBL database, https://www.ebi.ac.uk/chembl/ METAL (version released on 2011-03-25), https://github.com/statgen/METAL TwoSampleMR (v0.5.3), https://github.com/mrcieu/TwoSampleMR ggplot2 (v3.2.1), https://cran.r-project.org/web/packages/ggplot2/index.html coloc (v3.1 & v5.1), https://github.com/chr1swallace/coloc/tags epigraphdb (v0.2), https://epigraphdb.org/

## Abbreviations

AD: Alzheimer disease
BMD: Heel bone mineral density
BMI: Body mass index
CHD: Coronary heart disease
CSF: Cerebrospinal fluid
DBP: Diastolic blood pressure
dvt: Deep venous thrombosis
eQTL: Expression QTL
FDR: False discovery rate
FEV1: Forced expiratory volume in 1-s
FVC: Forced vital capacity
GTEx: Genotype-Tissue Expression
GWAS: Genome-wide association studies
IBD: Inflammatory bowel disease
IQR: Interquartile range
IV: Instrumental variable
IVW: Inverse variance weighted
LD: Linkage disequilibrium
MAF: Minor allele frequency
MR: Mendelian randomization
PC: Principal component
PP: Posterior probability
PPMI: Parkinson’s Progression Markers Initiative
pQTLs: Protein quantitative trait loci
QC: Quality control
RA: Rheumatoid arthritis
SBP: Systolic blood pressure
UC: Ulcerative colitis
WashU: Washington University
