# Supplementary material for: Mendelian randomization and genetic colocalization infer the effects of the multi-tissue proteome on 211 complex disease-related phenotypes
Source: Genome Med. 2022 Dec 12;14:140. doi: 10.1186/s13073-022-01140-9 (PMC9746220; doi:10.1186/s13073-022-01140-9)
Supplement: Supplementary file 1 — Additional file 1. Supplementary figures (Fig. S1–S8). [file 13073_2022_1140_MOESM1_ESM.pdf]

Mendelian randomization and genetic colocalization infer the effects of the multi-tissue proteome on 211 complex disease-related phenotypes

Chengran Yang, Anne M. Fagan, Richard J. Perrin, and Carlos Cruchaga*

FigureS1. Power analysis on the pQTL datasets.

FigureS2 Distribution of IVs overlapped with pleiotropic variants identified from all three tissues across different protein thresholds stratified by options of IVs used in MR analysis.

FigureS3 Additional significant protein-phenotypes associations identified using cis only study-wide pQTLs as instrumental variables after meta-analyses.

FigureS4 Additional significant protein-phenotypes associations identified using cis and trans genome-wide pQTLs as instrumental variables after meta-analyses.

FigureS5 Phenotype-category proportions of MR analyses from each tissue using only the 411 common proteins passed QC in all three tissues.

FigureS6 Enrichment of phenome-wide MR of the CSF, plasma, and brain proteome with the druggable genome.

FigureS7 Ranks of proteins by the number of convincing protein-phenotype associations from MR and colocalization analyses.

FigureS8 Genetic correlation of phenotypes with top ranked protein from MR and colocalization analyses.

Supplemental Figures

Tissue	pQTL effect size median	Protein-level sigma median
CSF-WashU	Cis: 0.072<br>Trans: 0.044	0.11
Plasma-WashU	Cis: 0.093<br>Trans: 0.068	0.119
Brain-WashU	Cis: 0.107<br>Trans: 0.038	0.084

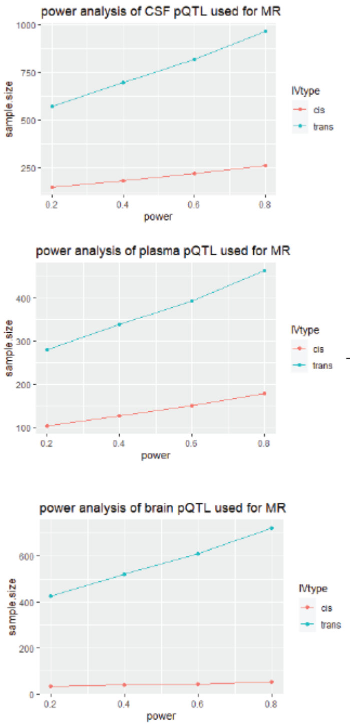

Tissue	Power given median beta
CSF-WashU [n = 835]	Cis: 1<br>Trans: 0.63
Plasma-WashU [n = 529]	Cis: 1<br>Trans: 0.91
Brain-WashU [n = 380]	Cis: 1<br>Trans: 0.42

FigureS1 Power analysis on the pQTL datasets. Effect size and variance were first learned from the pQTL used in MR analyses from each tissue. The relationship between power and sample size were next derived and visualized. The corresponding power given the sample size in the current study were finally calculated.

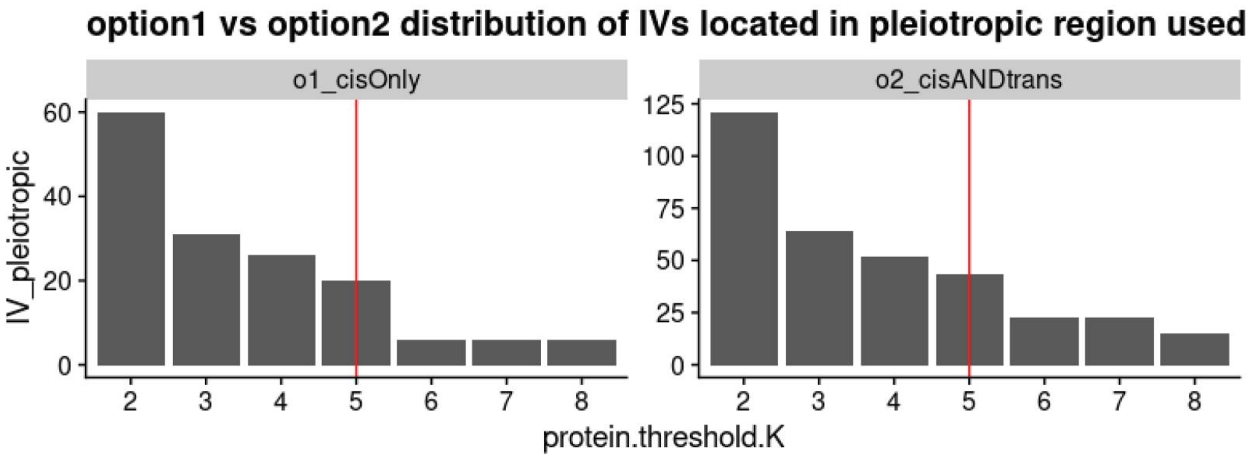

FigureS2 Distribution of IVs overlapped with pleiotropic variants identified from all three tissues across different protein thresholds stratified by options of IVs used in MR analysis. Number of genetic variants used as instrumental variables overlapped with pleiotropic variants identified from all three tissues varying from seven different protein thresholds (from 2 to 8) as empirical pleiotropic regions.

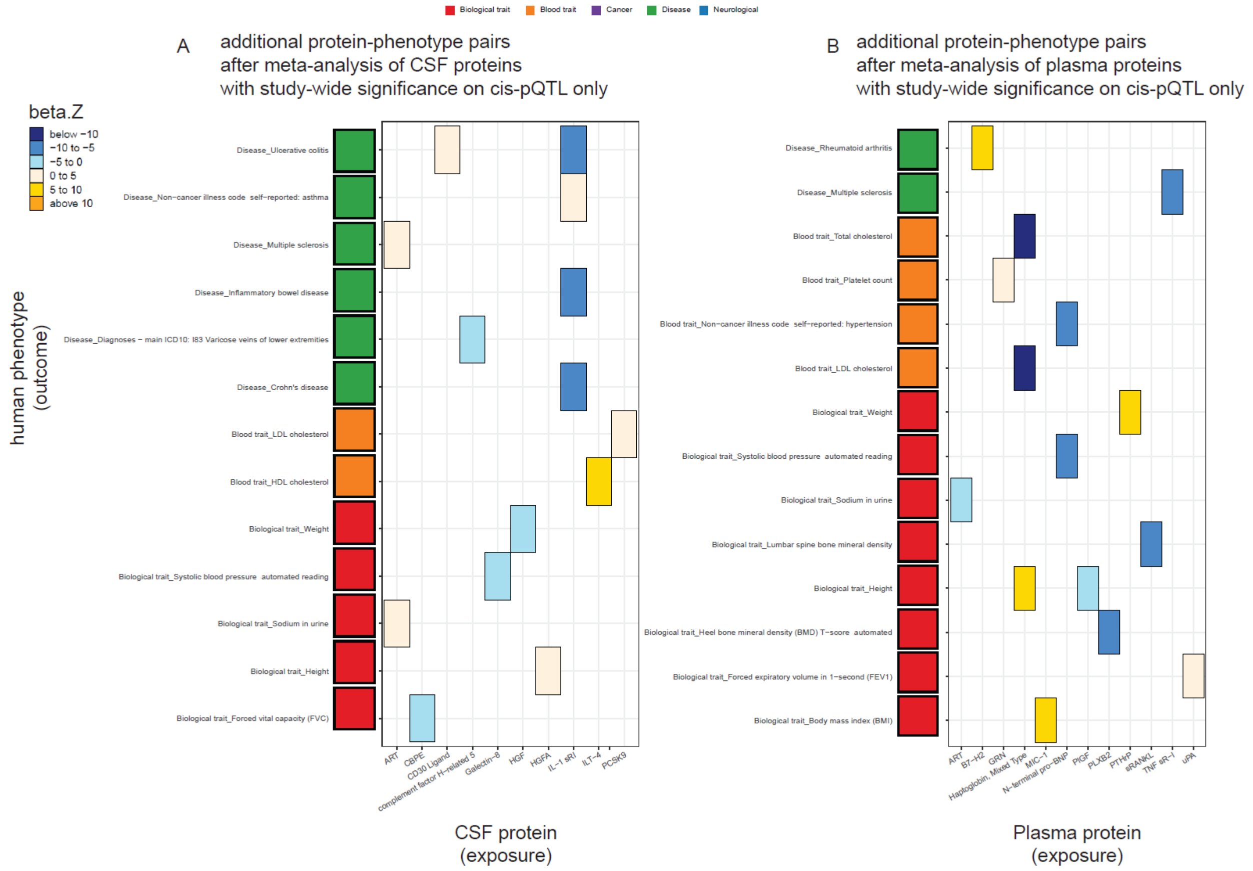

FigureS3 Additional significant protein-phenotypes associations identified using cis only study-wide pQTLs as instrumental variables after meta-analyses. Heatmaps were generated using the results of meta-analyses. **A)** 10 proteins on 13 diseases in CSF; **B)** 12 proteins on 14 diseases in plasma. Colors were coded by 6 bins after cutting z-normalized beta MR estimate: below -10 as darkblue; -10 to -5 as dodgerblue; -5 to 0 as cadetblue1; 0 to 5 as antiquewhite1; 5 to 10 as gold; above 10 as orange. Phenotype categories were listed on the left side as a bar-plot (biological traits as red, blood traits as orange, cancers as purple, non-neurological diseases as green, neurological diseases as blue).

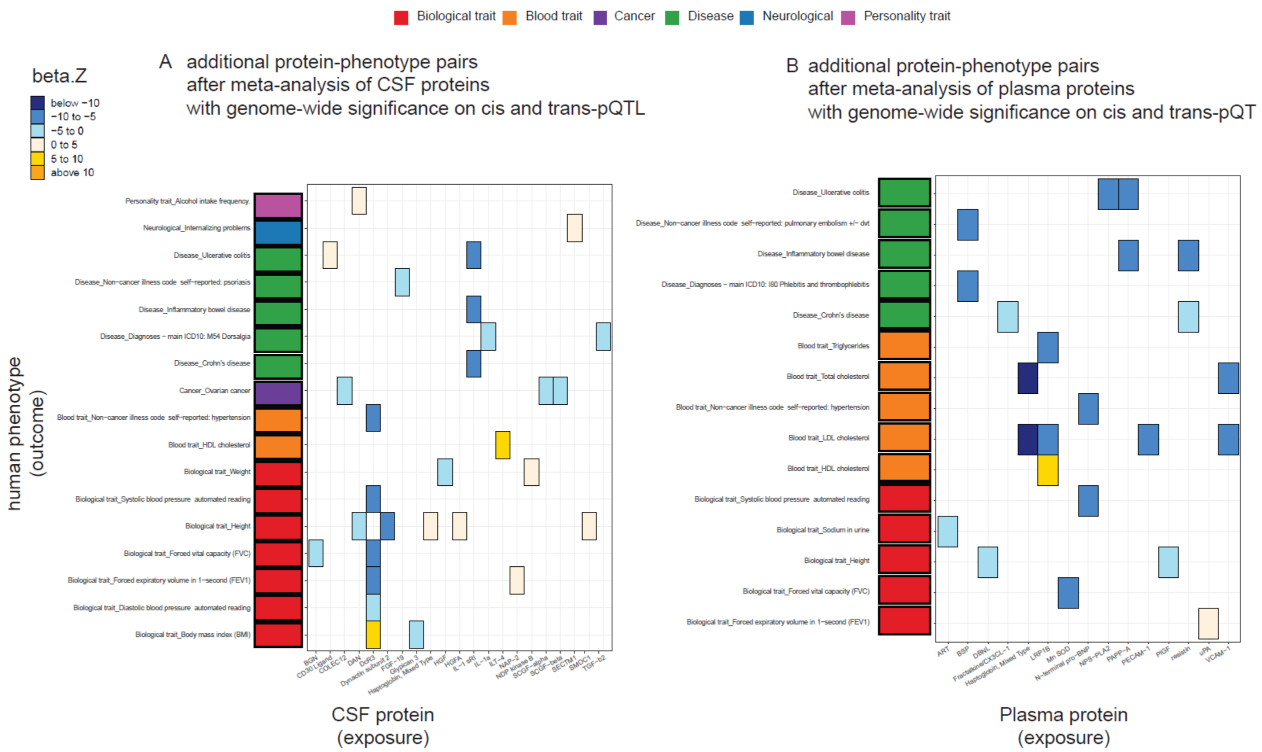

FigureS4 Additional significant protein-phenotypes associations identified using cis and trans genome-wide pQTLs as instrumental variables after meta-analyses. Heatmaps were generated using the results of meta-analyses. **A)** 21 proteins against 17 diseases in CSF; **B)** 15 proteins against 15 diseases in plasma. Colors were coded by 6 bins after cutting z-normalized beta MR estimate: below -10 as darkblue; -10 to -5 as dodgerblue; -5 to 0 as cadetblue1; 0 to 5 as antiquewhite1; 5 to 10 as gold; above 10 as orange. Phenotype categories were listed on the left side as a bar-plot (biological traits as red, blood traits as orange, cancers as purple, non-neurological diseases as green, neurological diseases as blue, personality traits as pink).

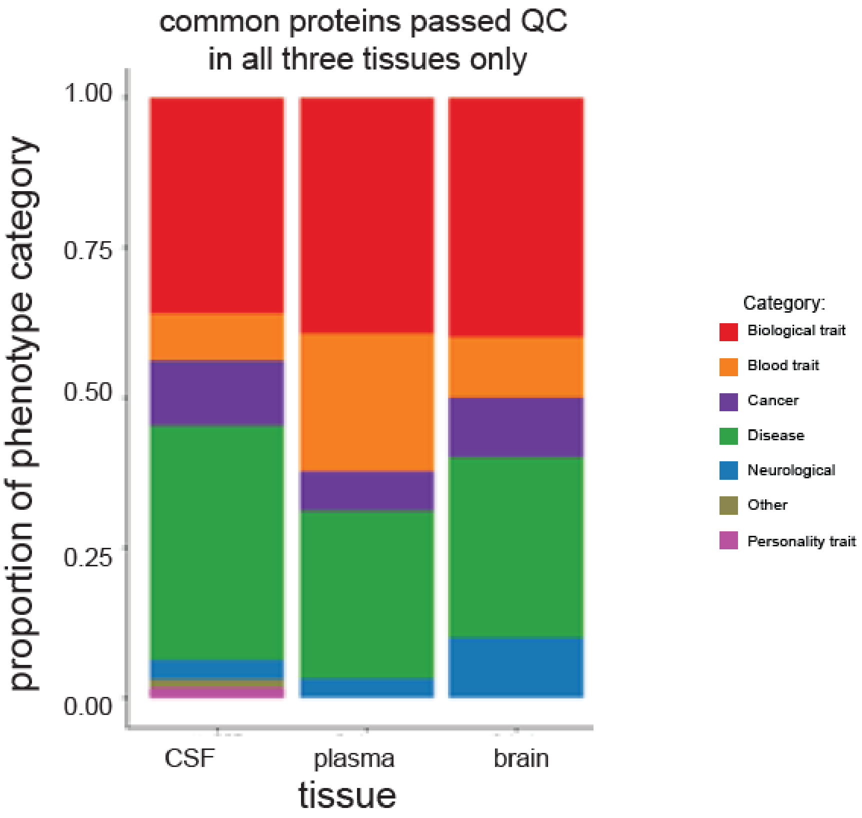

FigureS5 Phenotype-category proportions of MR analyses from each tissue using only the 411 common proteins passed QC in all three tissues. Barplots were used to visualize proportions of phenotype category per tissue and percentage of each proportion was listed in the table in parallel. The MR results are from combined analyses; Phenotype categories were color coded as biological traits as red, blood traits as orange, cancers as purple, non-neurological diseases as green, neurological diseases as blue, personality traits as pink, other risk factors as khaki.

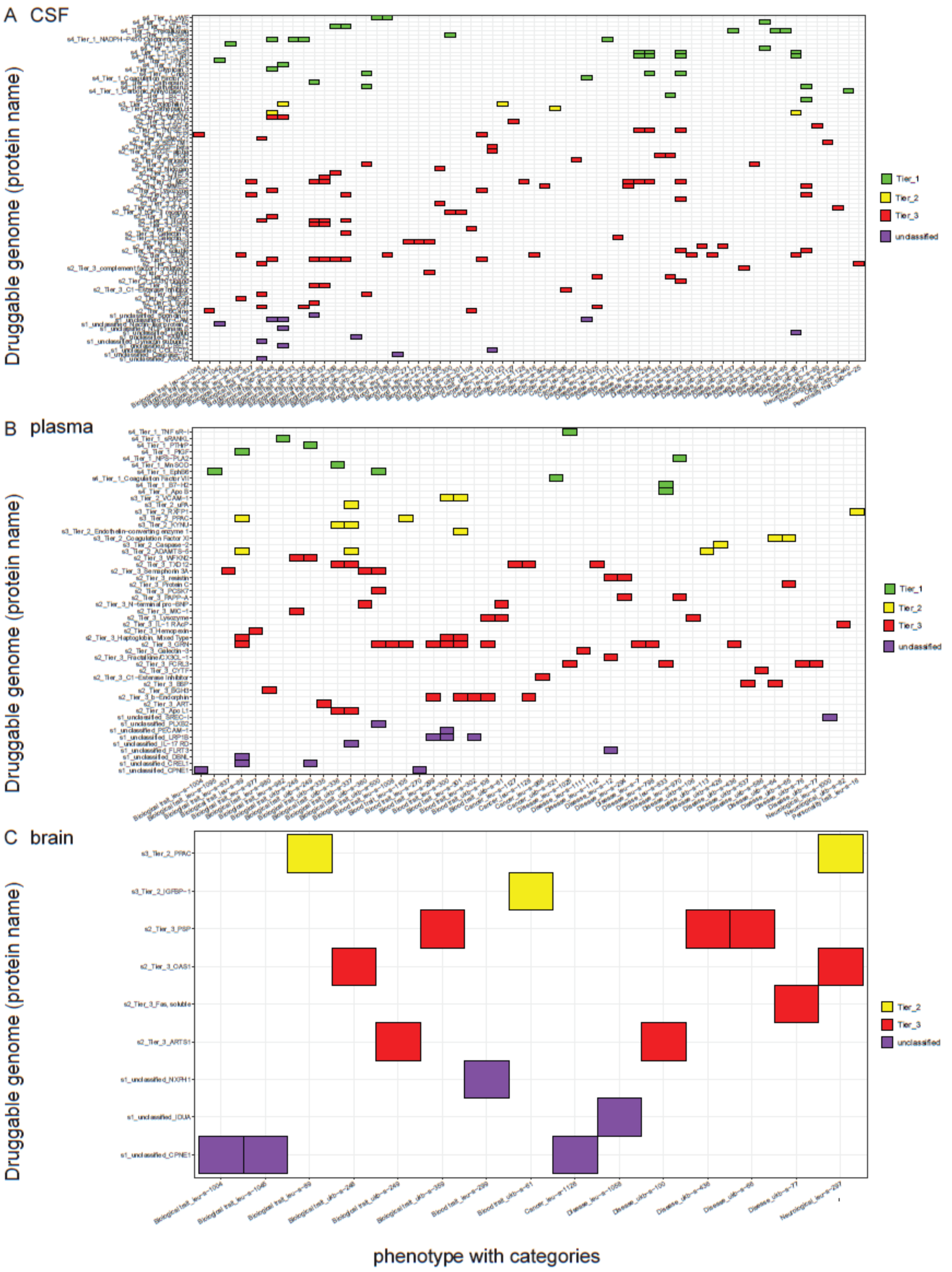

FigureS6 Enrichment of phenome-wide MR of the CSF, plasma, and brain proteome with the druggable genome. Heatmap was used to visualize the druggable-genome against categories of phenotypes on proteins with convincing MR and colocalization evidence, as illustrated by Zheng and colleagues[7]. There were seven major categories: 1) biological trait, 2) blood trait, 3) cancer, 4) non-neurological disease, 5) neurological disease, 6) personality trait, and 7) other phenotypes. The Y-axis presents the tiers of the druggable genome (as defined by

Finan and colleagues) of proteins under analysis in A) CSF; B) plasma and C) brain, with proteins classified into four groups based on their druggability: tier-1 contains proteins that are efficacy targets of approved small molecules and biotherapeutic drugs; tier-2 contains proteins closely related to approved drug targets or with associated drug-like compounds; tier-3 contains proteins distantly related to approved drug targets; and proteins have unknown druggable status (unclassified). The colored cells denote protein–phenotype associations with convincing MR and colocalization evidence. Cells from the heatmap in green, yellow, red, or purple represent associations with tier-1, tier-2, tier 3 or unclassified druggable genomes, respectively. The details can be found in Table S15.

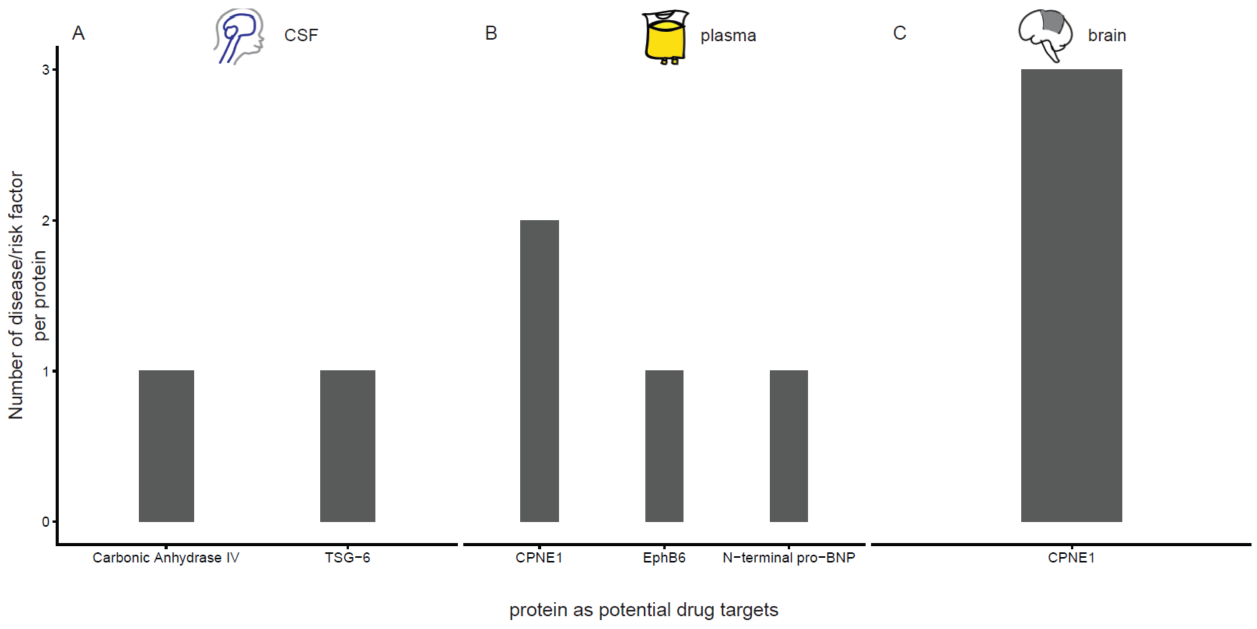

FigureS7 Ranks of proteins by the number of convincing protein-phenotype associations from MR and colocalization analyses. Bar plots of number of human phenotype (outcome) per protein (exposure). **A)** Two CSF proteins were ranked; **B)** Three plasma proteins were ranked; **C)** One brain protein was ranked.

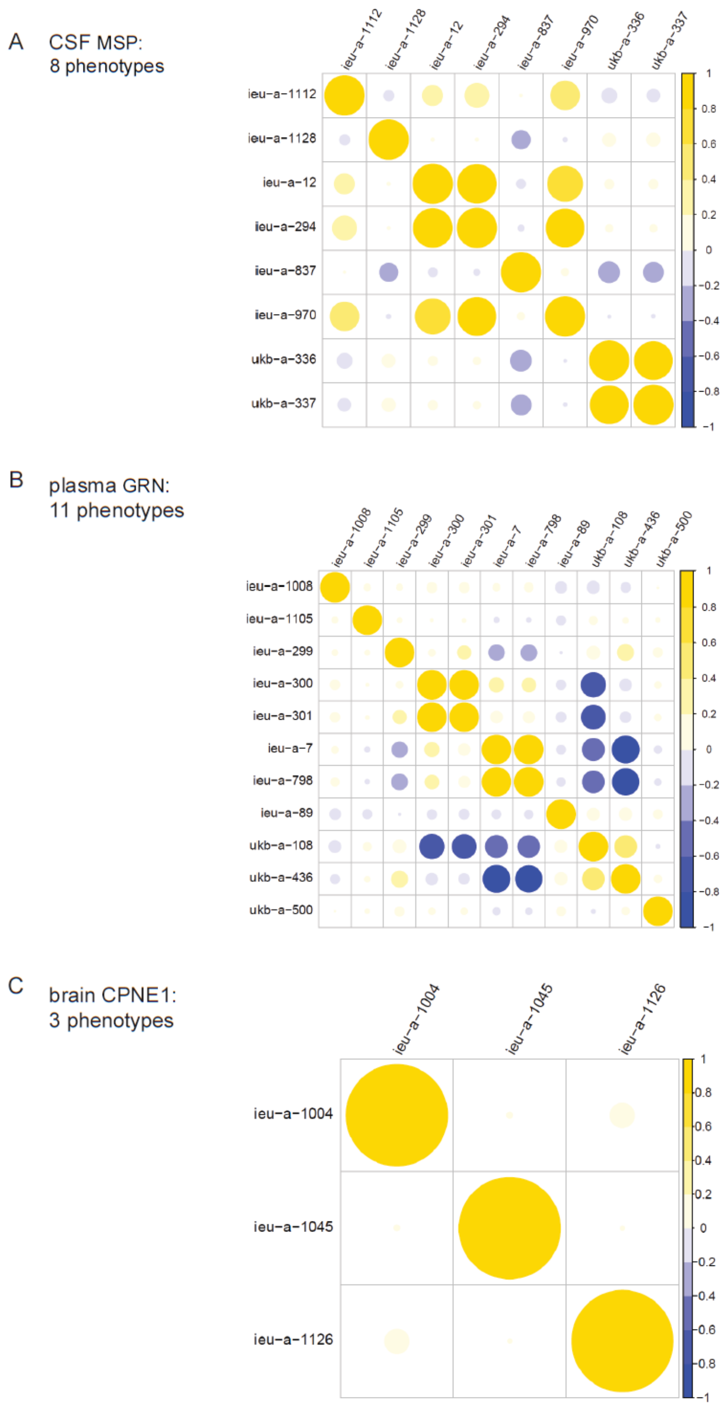

FigureS8 Genetic correlation of phenotypes with top ranked protein from MR and colocalization analyses. Heatmap was used to visualize the phenotype-phenotype correlation matrix of the top ranked protein within each tissue: **A)** CSF MSP associated with eight phenotypes; **B)** plasma GRN associated with 11 phenotypes; **C)** brain CPNE1 associated with three phenotypes. Blue represents a negative correlation; yellow represents a positive correlation.
